# T36. THE ANTIPSYCHOTIC-LIKE PROPERTIES OF EVENAMIDE (NW-3509) REFLECT THE MODULATION OF GLUTAMATERGIC DYSREGULATION

**DOI:** 10.1093/schbul/sby016.312

**Published:** 2018-04-01

**Authors:** Marco Bortolato, Laura Faravelli, Ravi Anand

**Affiliations:** 1 University of Kansas; 2 Newron Pharmaceuticals S.p.A; 3 Anand Pharma Consulting

## Abstract

**Background:**

The lack of adequate benefit with current 5HT2 / D2 antipsychotics in large proportions of schizophrenic patients suggests it is essential to modulate other mechanisms for improving symptoms of schizophrenia (SCZ). Increasing evidence implicates NMDAr hypofunction, and hippocampal hyperactivity, in the dysregulation not only of mesolimbic DA neurons but also of Glu neurons, leading to increasing synaptic activity of Glu in the PFC. Injection of NMDAr antagonists (PCP, ketamine) at doses that produce psychotomimetic effects lead to a downstream increase of Glu neurotransmission at non-NMDAr. The excessive firing and the hyper-glutamatergic tone represent alternative targets of treatment for SCZ ultimately affecting positive, negative, cognitive symptoms. The addition of Glu release inhibitors may augment the benefits of the antipsychotics in patients showing inadequate response.

Evenamide uniquely does not interact with monoaminergic (DA, 5-HT, NA, H) pathways affected by current antipsychotics, or with more than 130 different targets that are involved in CNS activity, except sodium channels. Preclinical data suggests that by the modulation of the firing abnormalities, evenamide normalizes the aberrant spread of Glu excitatory transmission that occurs in the brains of patients with SCZ. Evenamide showed efficacy in animal models relevant to SCZ (sensory motor gating, mania, psychosis, depression, impulse control, cognition, social interaction), in monotherapy and as an add on to first or second generation antipsychotics irrespective of whether impairment was either spontaneous, induced by amphetamine or NMDAr antagonists or stress. Evenamide, has also shown significant benefit in a p.c phase 2 trial as an add-on to risperidone and aripiprazole in patients worsening on dopaminergic/serotoninergic antagonist medication, suggesting it acts through other mechanisms. New animal data further confirm evenamide’s activity in reducing SCZ symptoms provoked by Glu alteration.

**Methods:**

Effects of evenamide (EVE 1.25, 5, 15 mg/kg PO) to restore the impaired information processing (a deficit observed in SCZ), were evaluated in the rat model of the Pre-Pulse Inhibition (PPI) deficit induced by injection of the NMDAr antagonist ketamine (KET 6 mg/kg, SC). Clozapine (CLO 7.5 mg/kg, IP) was used as a positive control.

**Results:**

PPI analysis showed significant main effects for KET to lower PPI levels [F(1,264)=139.67, P<0.0001], for EVE [F(3,264)=3.14, P<0.05] and CLO to enhance PPI levels [F(1,98)=30.89, P<0.001]. Notably, significant EVE x KET [F(3,264)=2.79, P<0.05] and CLO x KET interactions [F(1,98)=5.45, P<0.05] were found.

Post-hoc analyses (Tukey’s) revealed that KET significantly lowered PPI (P<0.0001) for each group; both EVE (5 mg/kg) and CLO significantly increased PPI in KET-treated rats (P=0.02; p<0.001).

**Discussion:**

Evenamide as monotherapy has similar effect to clozapine in reversing ketamine- induced worsening of PPI. Together with previously demonstrated effects to reverse PCP-induced PPI and social interaction deficits, this further supports its potential to affect both positive and negative symptoms of SCZ by targeting altered Glu transmission.

Efficacy of evenamide as an add-on to antipsychotics would revolutionize development of novel antipsychotics that would target aberrant firing and Glu transmission in SCZ. Two clinical trials have been planned to support the hypothesis that the addition of evenamide should add a non-dopaminergic mechanism for augmenting antipsychotic efficacy in patients who are not responding adequately to current antipsychotic, and in patients with treatment resistant SCZ who are not responding/worsening on clozapine.

